# Expression of FGD4 positively correlates with the aggressive phenotype of prostate cancer

**DOI:** 10.1186/s12885-018-5096-9

**Published:** 2018-12-17

**Authors:** Alexia Bossan, Richard Ottman, Thomas Andl, Md Faqrul Hasan, Nupam Mahajan, Domenico Coppola, Ratna Chakrabarti

**Affiliations:** 1 Burnett School of Biomedical Sciences, University of Central Florida, Orlando, Florida USA; 2 Department of Surgery, Washington University in St Louis, St Louis, MO USA; 3 Department of Anatomic Pathology and Tumor Biology, Moffitt Cancer Center, Tampa, Florida USA

**Keywords:** Rho GEF, Prostate cancer, Cell cycle, Cell migration, Drug sensitivity

## Abstract

**Background:**

FGD4 (Frabin) is an F-actin binding protein with GTP/GDP exchange activity specific for CDC42. It is involved in reorganization of the actin cytoskeleton, which requires both actin binding and CDC42 activating function of FGD4. Expression of FGD4 is altered in patients with heterogeneous hereditary motor and sensory neuropathies as a result of demyelination of peripheral nerves.

**Methods:**

In this study, we examined the expression of FGD4 in prostate cancer specimens using immunohistochemistry and studied the function of FGD4 in maintaining cell phenotype, behavior and drug sensitivity using overexpression and siRNA-based silencing approaches. We used Mann-Whitney test for comparative analysis of FGD4 expression.

**Results:**

Our results show that the expression of FGD4 is upregulated in cancerous prostates compared to the luminal cells in benign prostatic hyperplasia, although the basal cells showed high staining intensities. We noted a gradual increase in the staining intensity of FGD4 with increasing aggressiveness of the disease. Inhibition of expression of FGD4 using siRNAs showed reduced proliferation and cell cycle arrest in G2/M phase of androgen dependent LNCaP-104S and androgen refractory PC-3 cells. Inhibition of FGD4 also resulted in reduced cell migration and CDC42 activities in PC-3 cells whereas, ectopic expression of FGD4 induced cell migration, altered expression of mesenchymal and epithelial markers and activation of CDC42/PAK signaling pathway. Reduced expression of FGD4 improved sensitivity of LNCaP-104S cells to the anti-androgen drug Casodex and PC-3 cells to the microtubule stabilizing drug docetaxel.

**Conclusions:**

Our data demonstrate a tumor promoting and a cell migratory function of FGD4 in prostate cancer cells and that inhibition of FGD4 expression enhances the response for both androgen-dependent and independent prostate cancer cells towards currently used prostate cancer drugs.

**Electronic supplementary material:**

The online version of this article (10.1186/s12885-018-5096-9) contains supplementary material, which is available to authorized users.

## Background

FGD4, a guanine nucleotide exchange factor (GEF) specific for CDC42 Rho GTPase, is an F-actin binding protein [1] which is essential for maintenance of myelination in Schwann cells [2]. The FGD4 gene belongs to a family of GEFs which includes FGD1, FGD2, FGD3, FGD5, FGD6 and FRG. Each contain an FYVE domain in addition to a Dbl homology (DH) domain for catalytic activity and two pleckstrin homology (PH) domains for targeting proteins to the membrane through phosphoinositide binding [3–5]. The PH1 domain helps the DH domain with its catalytic activity through binding of nucleotide free small G-proteins [6, 7]. The FYVE finger domain is responsible for membrane trafficking and phosphoinositide metabolism through interaction with phosphoinositol-3-phosphate (PI3P) [8]. All members of the FGD family show significant sequence homology within DH, PH and FYVE domains, but FGD4 has an additional actin binding FAB domain at the 5′ end which is not present in other FGD proteins [9]. The DH domain and the PH1 domains are responsible for activation of CDC42 through GTP exchange for GDP, whereas the FYVE domain and the PH2 domains are involved in indirect activation of Rac [10]. The functions of FGD4 are multifaceted and includes bundling of F-actin through F-actin binding with FAB domains, activation of Rho GTPase signaling pathway through increased concentration of GTP bound CDC42, and plasma membrane association through FYVE domain binding with phosphoinositides and PI3P. FGD4 domain structures suggest its role as a cross linker between membrane structure and actin cytoskeleton. Loss of function mutations in the FGD4 coding sequence leading to expression of truncated FGD4 causes motor-sensory neuropathies or autosomal recessive Charcot-Marie-Tooth (CMT) disease type 4 [2, 11, 12]. The effect of mutation is mediated through inhibition of guanine nucleotide exchange function leading to reduced CDC42 activity, which results in demyelination of peripheral nerves [13].

RhoGEFs are known to be involved in cancer progression through activation of G-proteins, including members of Rho GTPases. A number of RhoGEFs are overexpressed in prostate cancer, including Rac1 GEF P-Rex [14] and Vav3 GEF for RhoA, Rac and CDC42 [15, 16], and are responsible for promoting metastasis and castration resistant prostate cancer (CRPC) by regulating androgen receptor (AR) activity. Activated CDC42 promotes cell proliferation through its interaction with the effector proteins MLK [17] and ACK1/TNK, which activates ERK and AKT. Additionally, activated CDC42 modulates cell migration through interaction with PAK4 [18] and MRCK [19], activation of LIMK 1/2 and reorganization of actin cytoskeleton [20]. Although the function of CDC42 is well documented, the involvement of CDC42 specific GEF FGD4 in prostate cancer has not been reported.

Prostate cancer accounts for one of the leading causes of cancer related deaths in the Western world [21]. Advances in detection methods including magnetic resonance imaging and prostate specific antigen (PSA) screening [22, 23] helped detection of prostate cancer at earlier stages and designing effective therapeutic strategies, such as surgical or radiation therapies or in some cases active surveillance [24, 25]. Although initially indolent and responsive to definitive treatments prostate cancer can become aggressive and present biochemical recurrence with elevated levels of PSA [26, 27]. For patients who experience dissemination of prostate cancer after localized therapy androgen deprivation therapy (ADT) is considered as the standard of care as monotherapy or in combination therapy with other agents [28, 29]. Nonetheless, resistance to ADT is common, which leads to development of CRPC [30, 31]. Our earlier studies showed an association between ADT, using AR antagonist Casodex, and upregulation of FGD4 in androgen sensitive prostate cancer cells [32]. In this study, we show a positive correlation of FGD4 expression with aggressiveness of prostate cancer and tumor promoting properties of FGD4 in prostate cancer.

## Methods

### Tissue microarray and immunohistochemistry

A prostate cancer tissue microarray block (TMAs) was constructed in the Tissue Core Facility at the Moffitt Cancer Center. The TMA contains 0.5 cm cores of de-identified tissues from 263 patients. The composition of tumor samples is presented in Table 1. The H&E slides of each sample was reviewed and the diagnosis confirmed by a pathologist (DC), before being included in the TMA. The area of interest was outlined by the pathologist for sampling and the TMA was constructed using a TMA Instrument (Tissue Arrayer, Beecher Instruments). A TMA section 3 μm in thickness was stained using an anti-FGD4 antibody (GeneTex NIN3). Dual staining of TMA was performed using the following protocol. Deparaffinized TMA was subjected to antigen retrieval in citrate buffer (pH 6.0) in a pressure cooker for 45 mins. Next, the TMA was blocked by peroxidase (3%) blocking solution followed by goat serum. Primary antibody was applied and incubated at room temperature for 1 h. Anti-rabbit secondary antibodies (Dako’s Envision anti rabbit RTU polymer system) were applied next and incubated at room temperature. Next, ImmPACT™ DAB substrate (Vector Laboratories) was added, incubated at room temperature, washed, and counterstained with SelecTec hematoxylene (Leica). The stained TMA was imaged using Aperio AT2 digital whole slide scanner (Leica) and evaluated using ImageScope image analysis software. The staining intensity analysis and the scoring was also done by a pathologist. The immunostaining was scored using the Allred scoring system [33].

**Table 1:** Compostition of the prostate TMA

Diagnosis	No. Cores	Diagnosis	No. Cores
BPH	23	GS7	39
PIN-Lo	24	GS 8, 9, 10	51
PIN-Hi	15	M	10
GS6	32	AI (GS 7–10)	13

### Cell lines and transfection

PC-3 cells obtained from ATCC were cultured in F12 HAM supplemented with 10% fetal bovine serum (FBS) and 1% antibiotic/antimycotic (Invitrogen). The androgen dependent LNCaP-104S cells (a gift from Dr. Shutsung Liao, University of Chicago) were isolated from the parental LNCaP cells and characterized [34]. LNCaP-104S cells were maintained in DMEM containing 10% FBS, 1 nM DHT (Sigma-Aldrich) and 1% antibiotic/antimycotic. C4-2B cells are developed from vertebral metastasis of LNCaP xenografts [35] (a gift from Dr. Leland Chung, Cedars-Sinai Medical Center). C4-2B cells were maintained in RPMI with 10% FBS and 1% antibiotic/antimycotic. All cells are rigorously monitored for mycoplasma contamination using DAPI staining method and authenticated through STR profiling (Additional file 1: Table S1). For inhibition of FGD4 expression, 4 FGD4 siRNAs (FlexiTube Gene Solution, Qiagen) were used for transient transfection using Lipofectamine RNAiMax (ThermoFisher) transfection reagent. Three control siRNAs were used for transient transfection in parallel as the controls. Transfected cells were harvested at 48 h for subsequent experiments. For ectopic expression, DNA of full length coding sequence of *FGD4* gene was synthesized (GenScript), cloned into pcDNA 3.1 and pcDNA 3.1-EGFP mammalian expression vectors (FGD4 pcDNA and pcDNA 3.1 FGD4-EGFP) and sequence verified. FGD4 pcDNA, pcDNA 3.1 FGD4-EGFP, pcDNA 3.1 MECP2-EGFP, or the empty vector as the control, was used for transient transfection using Lipofectamine (Invitrogen). Cells were used after 48 h for subsequent experiments.

### RNA extraction and quantitative real-time PCR

Total RNA from transfected cells was extracted using RNeasy kit (Qiagen). Total RNA was converted to cDNAs using QuantiScript Reverse Transcriptase (Qiagen) and used for quantitative PCR using FGD4 QuantiTect forward and reverse primers (Hs_FGD4_1_SG QuantiTect, Qiagen). The primers were designed to provide maximum efficiency for relative quantification. Quantitative PCR was conducted using Rotor-Gene SYBR Green PCR reagents (Qiagen) and Qiagen Rotor-Gene Q thermal cycler and data analyzed using Rotor-Gene-Q software. DNA concentration was assessed using SYBR Green fluorescence and C_t_ values generated were normalized using C_t_ values of RPL13A and GAPDH normalizer genes. The C_t_ values were used to derive ^ΔΔ^Ct values using the miRNome analysis software (System Biosciences).

### Western blotting

Lysates of transfected PC-3, LNCaP-104S and C4-2B cells were prepared and used for immunoblotting using anti-FGD4, anti-E-cadherin, anti-SLUG, anti-phospho PAK, anti-phospho cofilin, anti-GAPDH and anti-alpha-tubulin antibodies (Additional file 1: Table S2). Signals were detected using enhanced chemiluminescence (ECL) detection method. GAPDH and alpha-tubulin were used as the loading controls. Comparative analysis of the target protein expression was performed using densitometric analysis of the normalized peptide band intensity.

**Table 2:** p Values pairwise comparison of FGD4 expression between tumor categories

	PIN-Lo	PIN-Hi	G6	G7(3 + 4)	G7(4 + 3)	G8910	CRPC
BPH	0.09296	0.08364	0.00244	0.00544	0.00544	0.00138	0.00094
PIN-Lo		1.0	0.72634	0.68916	0.41222	0.09296	0.0601
PIN-Hi			0.70394	0.42952	0.79486	0.12852	0.03156
G6				0.50286	0.72786	0.04884	0.01078
G7(3 + 4)					0.71138	0.09296	0.06724
G7(4 + 3)						0.05238	0.07346
G8910							0.96012

### Cell proliferation and drug sensitivity assays

PC-3 and LNCaP-104S cells were seeded in 96 well plates and transfected with FGD4 siRNAs or control siRNAs after 24 h or 48 h after seeding. Transfection medium was replaced with fresh medium after 8 h of transfection. For drug sensitivity assays, the media were replaced with 10 μM Casodex or DMSO in 20% charcoal-stripped FBS (CS-FBS) containing growth medium (LNCaP-104S) or 5 nM and 25 nM Docetaxel, or the vehicle in regular complete growth medium (PC-3). Cell proliferation was detected at 48 h after transfection using MTS based Cell Titer Aqueous One Solution cell proliferation assay kit (Promega).

### Flow cytometry

PC-3 and LNCaP-104S cells were seeded in a 12-well dish and transfected with FGD4 siRNAs or control siRNAs after 24 h or 48 h. Cells were harvested at 48 h post transfection and resuspended in cold PBS before being placed on ice. Ice-cold methanol was added to fix and permeabilize the cells. The cells were left at -20 °C in methanol for 30 min. The tubes were returned to ice and cold PBS was added to the tubes. Cells were incubated on ice for an additional 5 min, centrifuged and rinsed with PBS twice and resuspended in PBS containing 50 μg/mL RNase and 2% Bovine Serum Albumin (BSA) in PBS. The tubes were incubated for 15 min at room temperature and then diluted with 2% BSA in PBS. Propidium iodide (PI) in 2% BSA in PBS solution was added to each tube to achieve 50 μg/ml and the tubes were incubated in the dark at room temperature for 1 h. The cells were run in a BD Accuri flow cytometer until a count of 10,000 PI-stained events was obtained per sample. FlowJo Analysis software was used for cell cycle analysis.

### Scratch assay

PC-3 and C4-2B cells were seeded into 24-well plate in growth medium containing 5% FBS and transfected with FGD4 siRNAs or control siRNAs, or FGD4 pcDNA or the control vector after 24 h of seeding. Transfection media was replaced with fresh media after 8 h and incubation continued for 48 h. Wounds in the form of a straight scratch were created using a micro pipet tip. Following the scratch, cells were rinsed twice with PBS and incubated in media containing 5% FBS at 37 °C. Migration within the scratch was imaged at 0 h and 14 h or 24 h using a Nikon eclipse TE200 inverted microscope coupled with Nikon elements F 2.20 software. The average width of the scratch was determined using Image J software. The relative rate of migration was determined through the analysis of the remaining width of the scratch at 14 h and subtracting that from the 0 h. Next, the ratio of the distance traversed by the FGD4 DNA or FGD4 siRNA transfected and control DNA or control siRNA transfected cells respectively was determined.

### CDC42 activation assay

PC-3 cells were seeded and grown to 70% confluence before being transfected with FGD4 siRNA or control siRNA. Cells were harvested at 48 h after transfection and subjected to CDC42 activation assay using CDC42 G-LISA Activation Assay kit (Cytoskeleton) according to the manufacturer’s protocol. Briefly, cell lysates were prepared and equal amounts of protein were added to the tubes containing GTP-binding protein and incubated with shaking at 4 °C. Unbound proteins were washed off and antigen presenting buffer was added in tubes and incubated at room temperature. Primary antibodies were added and incubated at room temperature. Next, tubes were washed and secondary antibodies were added to them. Samples were incubated at room temperature, washed and HRP detection reagent mix followed by HRP stop buffer were added and absorbance of the developed color was detected in a spectrophotometer at 490 nm.

### Dual label immunofluorescence

C4-2B cells were seeded in Permanox Lab-Tek chamber slides, and after 24 h transfected with either a FGD4-EGFP fusion construct, pcDNA-FGD4–3’EGFP, or a control fusion protein, pcDNA-MECP2-EGFP. After 24 h cells were treated with DMSO or ROCK inhibitor Y-27632 (20 μM) (Selleck Chemicals) or kept untreated. Cells were incubated for 24 h and fixed with 4% paraformaldehyde in PBS and permeabilized with 0.01% Triton-X-100. To visualize the actin cytoskeleton, cells were stained with 0.26 μM Alexa Fluor 647-phalloidin for 10 min (Cell Signaling). Cells were mounted with DAPI Fluoromount-G (Southern Biotech) and imaged using a Leica SP5 confocal microscope and images were analyzed using Leica LAS AF software suite.

### Statistical analysis

All statistical analyses were performed using Student t-test or one-way ANOVA for independent measures using GraphPad Prizm.

## Results

### Alteration of FGD4 expression in advanced prostate cancer

**Fig. 1:**
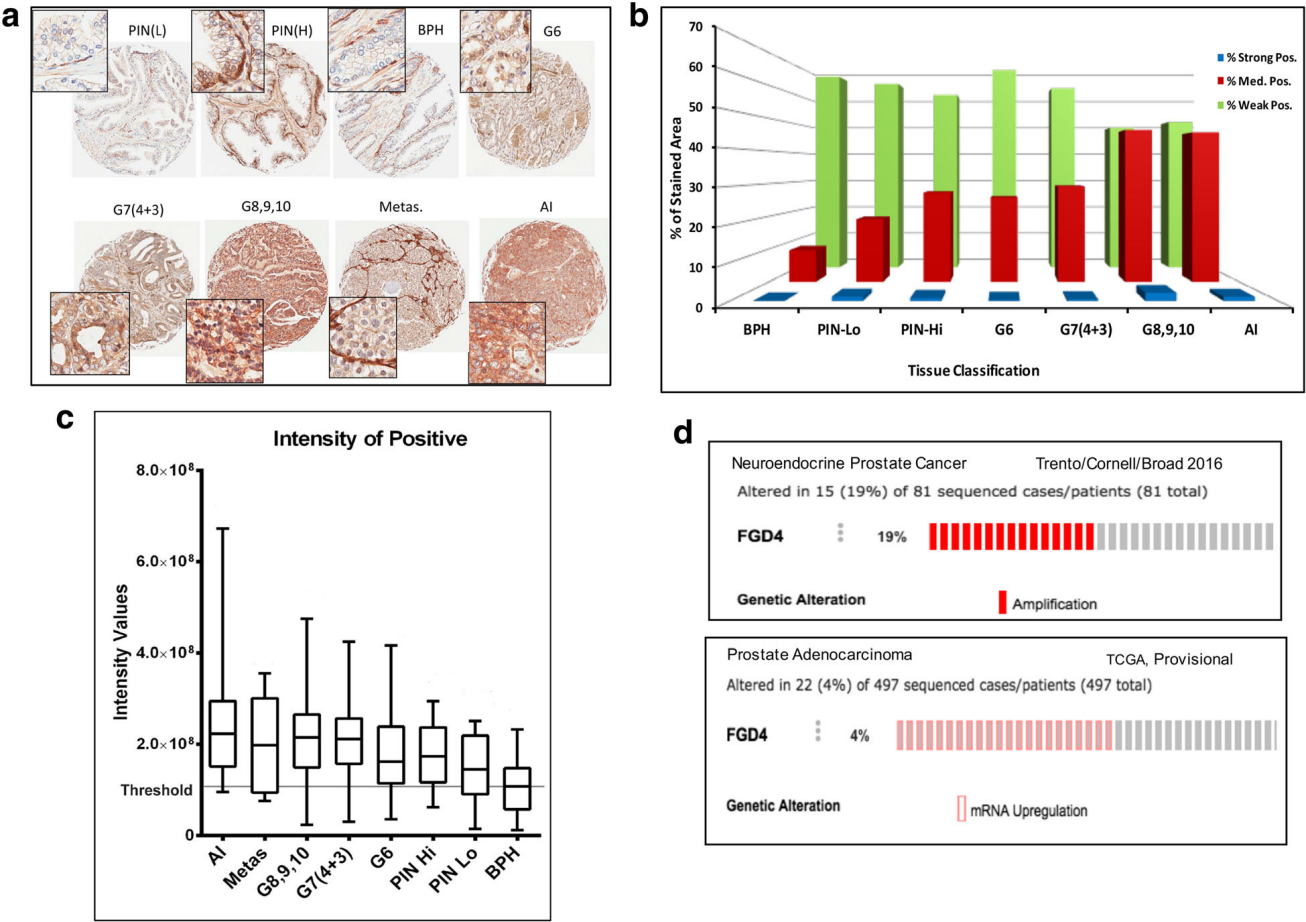
Expression of FGD4 in prostate tumors changes with disease progression. **a**: Representative TMA images of the immunohistochemical analysis of FGD4 expression in benign prostatic hyperplasia (BPH) and prostate tumor tissues of different Gleason Grades, metastatic stages, and castration resistant (AI) stages. Images show weak staining in the basal cells in PIN (L) while strong staining in BPH, PIN (HI) tissues. A moderately higher expression of FGD4 could be noted in the luminal cells of prostate tumors with Gleason Scores 6 and 7, whereas a strong expression is evident in prostate tumor exhibiting more advanced stages. **b**: Comparative analysis of the percentage of strong, medium and low positive staining areas in BPH and prostate cancer tissues. **c**: Comparative analysis of the staining intensity across different prostate tissues. **d**: TCGA database analysis showing alteration in FGD4 DNA copy number and mRNA expression in neuroendocrine prostate cancer and prostate adenocarcinoma.

Our previous studies on miRNA expression profiling during development of resistance to Casodex showed loss of expression of miR-17-92a cluster in drug resistant prostate cancer cells [32]. We also confirmed that FGD4 is one of the targets of miR-17 and -20a from this cluster and is upregulated in Casodex resistant prostate cancer cells [32, 36]. In this study, we monitored the expression pattern of FGD4 protein in prostate tumors using a custom tissue microarray (TMA) and immunohistochemistry. The TMA was generated using clinically annotated tissue samples from patients selected based on specific criteria. The tissue composition of the TMA includes benign prostatic hyperplasia (BPH), prostatic intraepithelial neoplasia low grade (PIN-Lo), prostatic intraepithelial neoplasia high grade (PIN-Hi), prostatic carcinomas (PCA) of different Gleason Scores, metastatic prostatic carcinoma (M) and castration resistant prostate carcinomas (AI) (Table 1). The cases in the TMA are both from biopsies and resections. Biopsy samples were used especially for the metastatic cases. Immunohistochemistry analysis revealed strong expression of FGD4 in basal cells but not in the luminal cells in BPH and in PIN (L) lesions (Fig. 1a and b). A moderately strong staining was noted in the basal cells and in the stromal areas in PIN (H) lesions (Fig. 1a and b), whereas a moderate to strong staining was noted in the cytoplasm of carcinomas. Occasionally, FGD4 nuclear stain was also noted in PCA with higher Gleason Scores and androgen independent status. Analysis of staining intensity showed a gradual increase in the median intensity of staining in tumors with higher Gleason scores and androgen independent status as compared to PCA of low Gleason score (6) and to BPH and PIN lesions (Fig. 1c). This observation revealed a positive correlation between FGD4 expression and progression of prostate cancer. Pairwise comparison of the *p* values using the Mann-Whitney test showed a significant difference in the FGD4 expression pattern between BPH, and PCA with Gleason Scores from 6 to 10 and CRPC tumors (Table 2), between PIN-Hi and CRPC tumors, and between PCA with Gleason Score 6, PCA with Gleason Scores 8–10 and CRPC tumors. Additionally, we performed TCGA database analysis of FGD4 copy number alteration and mRNA expression in prostate cancer using Trento/Cornell/Broad 2016 [37] and TCGA provisional datasets [38] (Fig. 1d), which revealed an increased FGD4 DNA copy number in 19% of the cases with neuroendocrine prostate cancer and mRNA upregulation in 4% of the cases with PCA (Fig. 1d).

### Knock down of FGD4 reduced cell proliferation and induced G2/M-arrest

**Fig. 2:**
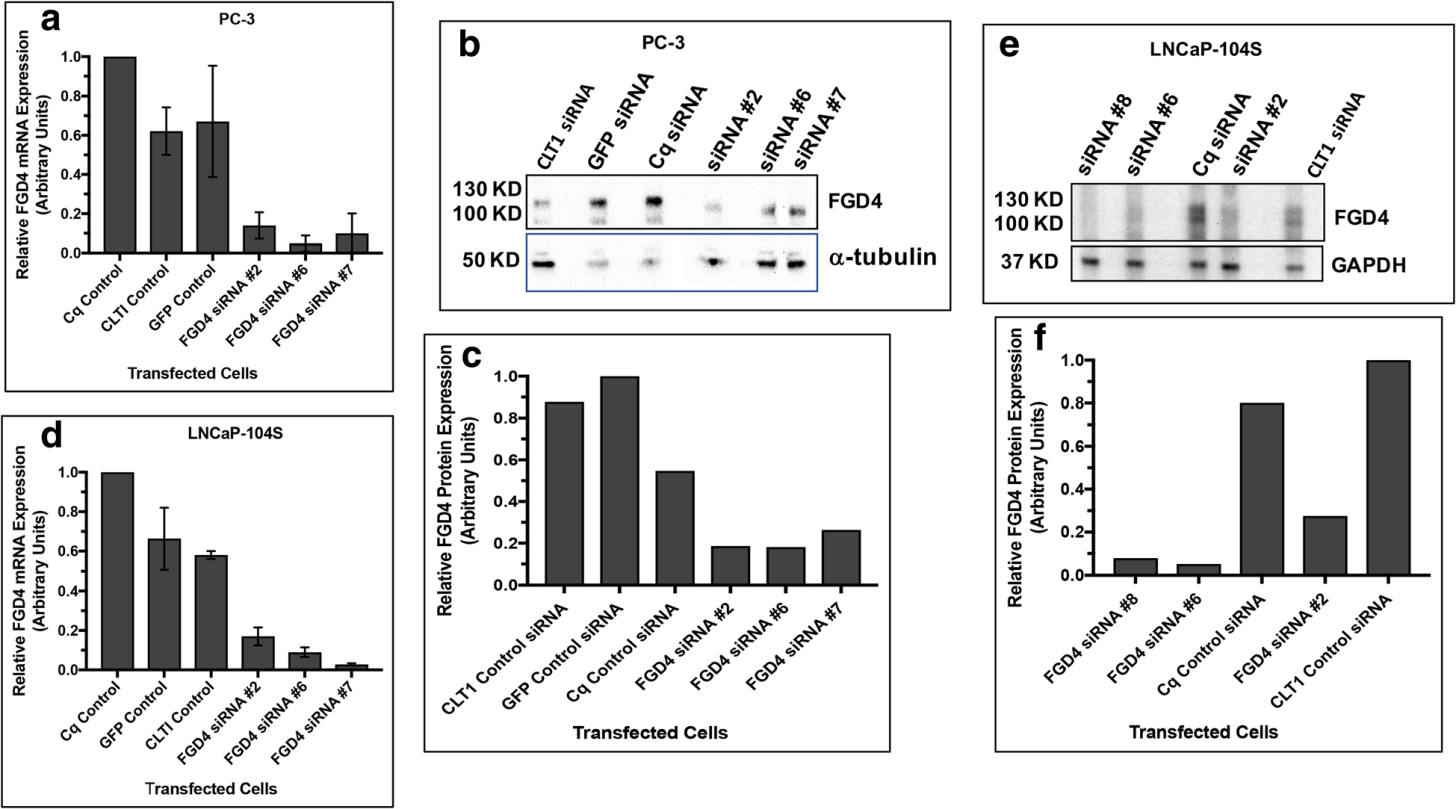
Inhibition of FGD4 expression upon FGD4 siRNA transfection of prostate cancer cells. **a** and **d**: Quantitative real-time PCR analysis of FGD4 mRNA in siRNA or control RNA transfected PC-3 and LNCaP-104S cells. Raw data have been normalized to the average of RPL13A and GAPDH, two control genes. Data show at least 70% reduction in FGD4 mRNA by FGD4 siRNAs compared to control siRNAs. **b** and **e**: Immunoblot analysis of PC-3 and LNCaP-104S cell lysates transfected with FGD4 siRNAs showing substantial reduction in FGD4 upon siRNA transfection. Alpha-tubulin and GAPDH were used as the loading controls **c** and **f**: Densitometric analyses of protein concentration normalized to the internal controls.

**Fig. 3:**
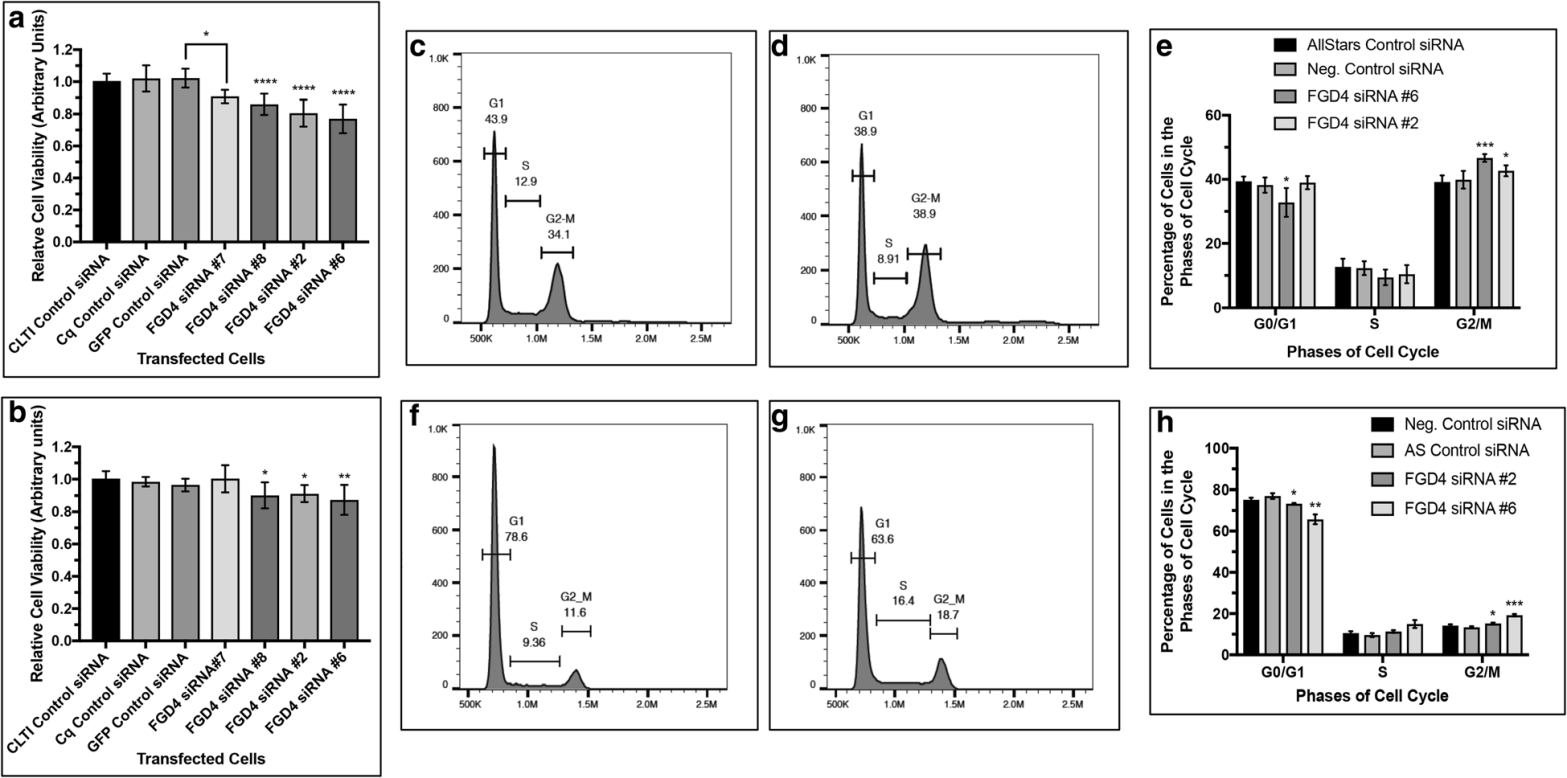
Inhibition of FGD4 decreased cell proliferation and arrested cells in G2/M phase. Cell proliferation assay showing a significant reduction in proliferation of PC-3 (**a**) and LNCaP-104S (**b**) cells transfected with two different FGD4 siRNAs compared to the control siRNAs. Data represent mean ± SD of at least three different experiments run in triplicate. **a**: * *p* < 0.0002, B: * *p* < 0.05, ** *p* < 0.005. **c**, **d**, **f** and **g**: Two parameter histogram showing a higher G2/M peak in siRNA transfected PC-3 (**d**) and LNCaP-104S (**g**) cells compared to control RNA transfected cells (**c** and **f**). **e** and **h**: Relative percentage of cells in different cell cycle phases upon inhibition of FGD4 expression in PC-3 (**e**) and LNCaP-104S (**h**) cells. Data represent mean ± SD of at least three independent experiments. **e**: *p < 0.05, *** *p* < 0.001 H: *p < 0.05, ** *p* < 0.01, *** *p* < 0.001).

To understand the functions of FGD4 in promotion of tumor cell growth and cell cycle progression we used FGD4 siRNAs for inhibition of FGD4 expression. We used two prostate cancer cell lines PC-3 and LNCaP-104S, which represent androgen independent and androgen dependent status of the prostate cancer and are developed from two different metastatic lesions viz. bone and lymph node. We used 4 different FGD4 siRNAs and three control siRNAs for our study. We used transiently transfected cells for analysis of FGD4 mRNA and protein reduction using RT-qPCR and Western blotting, respectively. Our results showed that of 4 FGD4 siRNAs, #2 and #6 were more effective (60–80% reduction) in inhibiting FGD4 mRNA and protein expression in both cell lines (Fig. 2). These two siRNAs were used for most of the subsequent experiments. Cell proliferation assays using MTS dye reduction showed a significant (10–23%) decrease in proliferation of PC-3 cells upon transfection with FGD4 siRNAs compared to control siRNAs (Fig. 3a). Knockdown of FGD4 in LNCaP-104S cells also showed a significant reduction in proliferation, though it was slightly less than the effects noted for PC-3 cells, with 8–13% reduction in proliferation compared to controls (Fig. 3b). Studies have shown that FGD4 participates in actin reorganization through direct interaction with F- actin [1]. Because actin reorganization is necessary for cell cycle progression, specifically the mitotic phase, next we analyzed the association of FGD4 on cell cycle progression using PI staining and flow cytometry. Our analysis revealed that knockdown of FGD4 induced G2/M arrest in both PC-3 and LNCaP-104S cells. (Fig. 3d, e, g, h) compared to control siRNAs (Fig. 3c, e, f, h). Cells transfected with FGD4 siRNAs showed a 20–40% increase in the G2/M phase and a decrease in number of cells in G1 phase compared to control siRNAs (Fig. 3e and h) in both cell lines. We also noted a 74% increase in doublets with 8 N chromosomes in PC-3 cells upon knockdown of FGD4 (data not shown). These results suggest that FGD4 activity is required for cell cycle progression.

### Knock down of FGD4 reduced cell migration and activation of CDC42

**Fig. 4:**
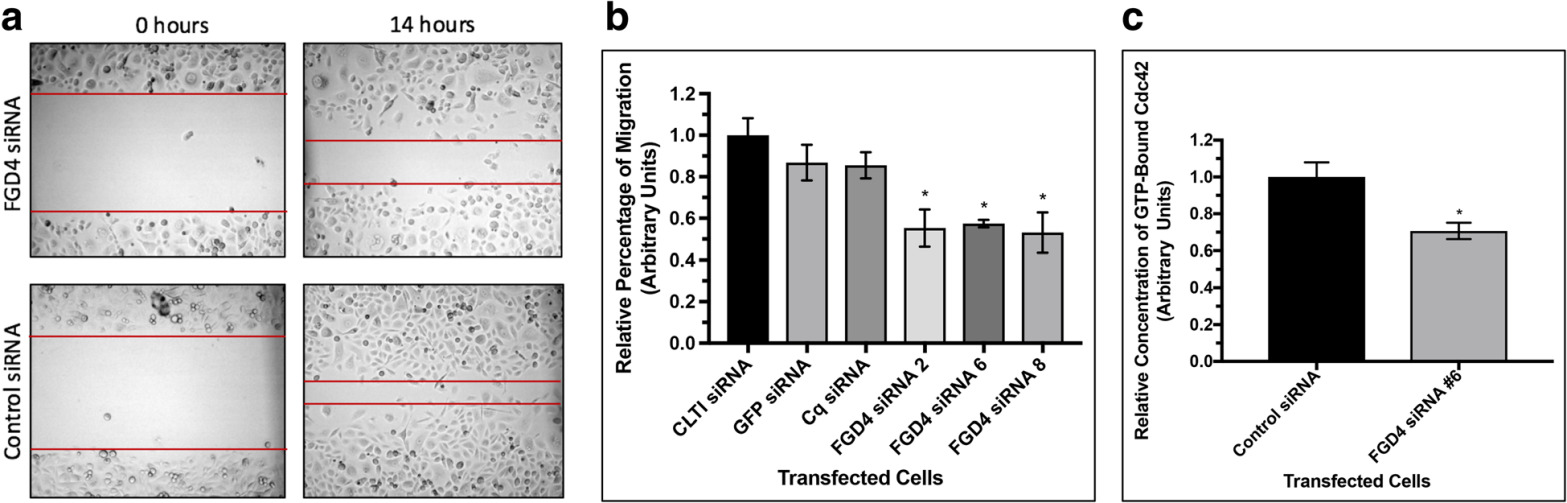
Inhibition of FGD4 expression reduced migration and activation of CDC42 in prostate cancer cells. **a**: Migration of PC-3 cells transfected with FGD4 siRNAs or control siRNAs. Migratory properties of PC-3 cells were tested through scratch assays at 0h and 14h after making the scratches. The width of each wound was measured at three areas using light microscopy. Three scratches were made in each dish and the experiment was conducted in triplicate. **b**: Relative rates of migration at 14h. Data presented as the ratio of the distance traversed by the PC-3 cells transfected with control siRNAs and FGD4 siRNAs. Data represent mean ± SD of three different experiments in triplicate scratches. *p<0.0001. **c**: Relative concentration of GTP-bound CDC42 in PC-3 cells transfected with FGD4 siRNA or control siRNAs. Data represent mead ± SD of three different experiments. *p<0.01

FGD4 also participates in maintenance of cell shape through activation of Rho GTPase CDC42, which led us to examine the effect of FGD4 knockdown on cell migration. We chose PC-3 cells for our study as these cells are highly migratory. We used scratch assays for monitoring the migration rate of PC-3 cells transfected with FGD4 siRNAs or the control siRNAs. The bright field images and quantification of the distance traversed by the transfected PC-3 cells 14 h after scratching showed a significant inhibition of migration of PC-3 cells upon knockdown of FGD4 using three different siRNAs (#2, #6 and #8) compared to control siRNA transfected cells (Fig. 4a). A nearly 40% reduction in cell migration was noted in FGD4 siRNA-transfected cells (Fig. 4b).

To further confirm that the inhibition of cell migration upon knocking down of FGD4 is associated with interference of the CDC42 activation, we examined the activation status of CDC42 by monitoring the levels of GTP-bound CDC42 in PC-3 cells upon transfection of FGD4 siRNA. We used FGD4 siRNA #6 which showed the maximum reduction in FGD4 mRNA and proteins for the activation assays. Our results showed a 30% reduction in GTP-bound CDC42 in cells with lower FGD4 concentration (Fig. 4c).

### Ectopic expression of FGD4 increased cell migration and expression of SLUG

**Fig. 5:**
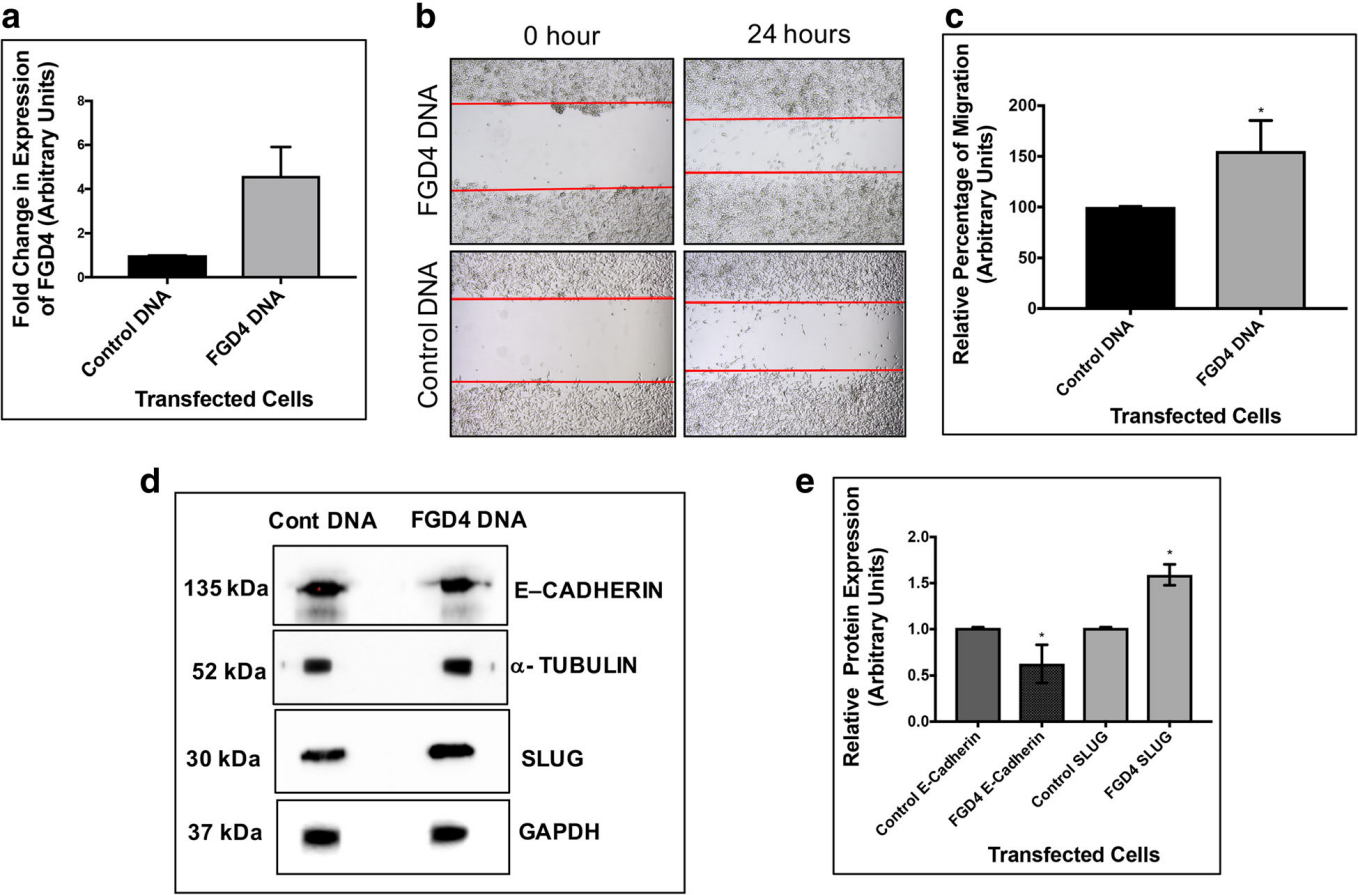
Overexpression of FGD4 promoted migration of prostate cancer cells and altered expression of markers of EMT. **a**: Quantitative real time PCR analysis of FGD4 mRNA in C4-2B cells transfected with *FGD4* pcDNA or the control vector. Expression of RPL13A and GAPDH mRNA were used to normalize FGD4 expression in transfected cells. Data show >3.0- fold increase in FGD4 expression in *FGD4* pcDNA transfected C4-2B cells compared to the control cells. **b**: Migration of C4-2B cells imaged at 24h after transfection with FGD4 expression vector or the empty vector. Four scratches were made in each dish and the experiment was conducted in triplicate. **c**: Relative rates of migration at 24h. Data represent mean ± SD of three different experiments in quadruplicate wounds. *p<0.03. **d**: Western blot analysis showing increased expression of expression of SLUG and decreased expression of Ecadherin in FGD4 transfected cells. **e**. Densitometric analysis of the expression of SLUG and E-cadherin using α-tubulin and GAPDH as the loading controls. Data show mean ± SD of two to three independent experiments. *p=0.005.

To confirm the involvement of FGD4 in prostate cancer cell migration, we used the approach of gene overexpression. We used C4-2B cells as these cells are moderately migratory, for ectopic expression of FGD4. A 4.5-fold increase in FGD4 mRNA was obtained in FGD4 pcDNA transfected cells compared to the empty vector transfected cells (Fig. 5a). Scratch assays showed a significant increase (55%) in migration of FGD4 expressing C4-2B cells at 24 h compared to the control cells (Fig. 5b and c). To understand the mechanism of FGD4 mediated cell migration we tested expression of SLUG, a mesenchymal marker [39] and E-cadherin, an epithelial marker [40] using Western blots. Our results showed a 40% reduction in E-cadherin expression and a 60% increase in SLUG expression in FGD4 over-expressing cells (Fig. 5d and e).

### FGD4 promoted activation of PAK and filopodia formation

**Fig. 6:**
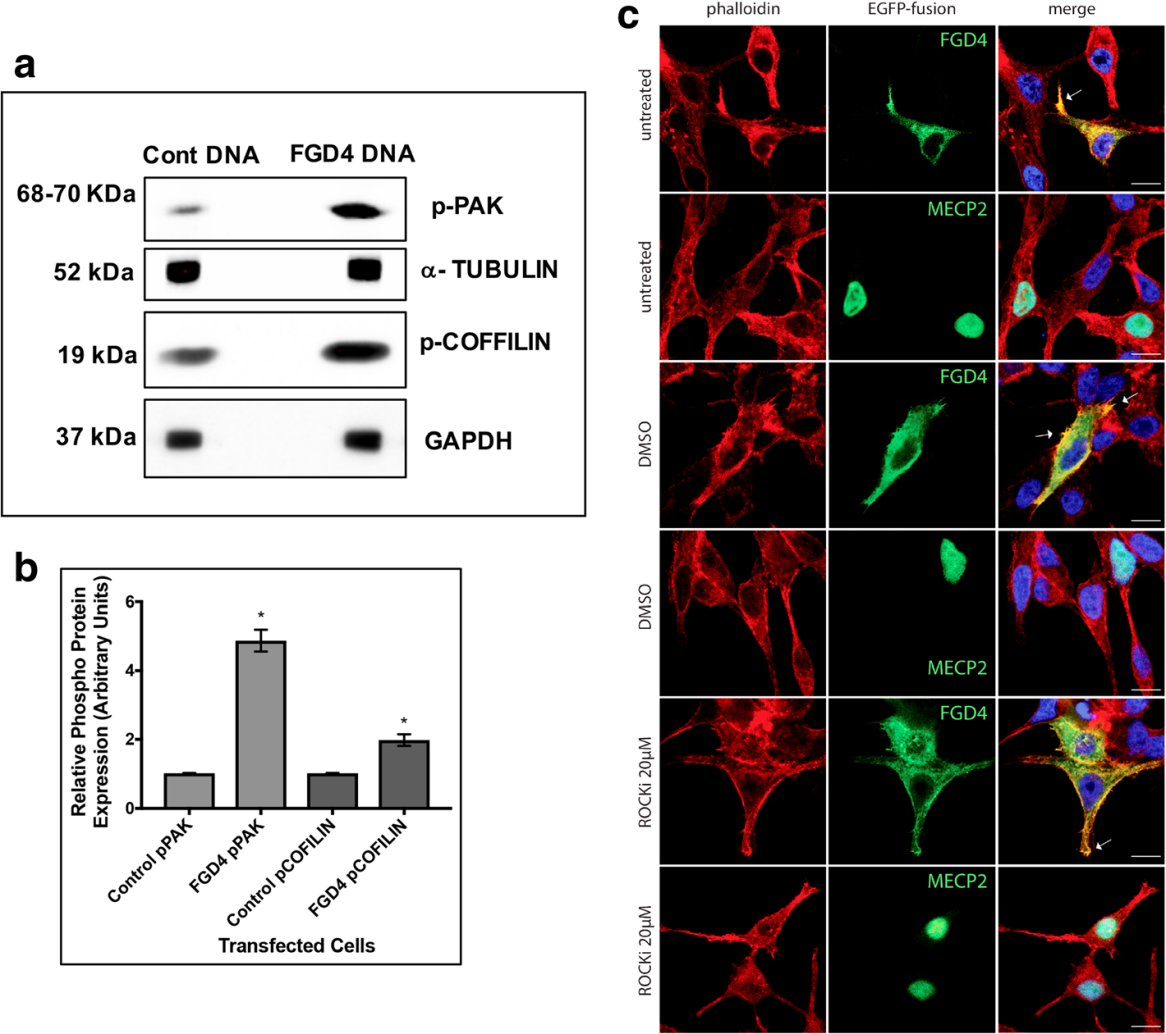
Overexpression of FGD4 induced phosphorylation of PAK and cofilin, and filopodia formation. **a**: Western blot analysis of phospho-PAK and phospho-cofilin in total extracts of C4-2B cells expressing FGD4 or an empty vector showing increased intensity of the peptide bands in FGD4 expressing cells. **b**: Densitometric analysis of phospho-PAK and phospho-cofilin expression using α-tubulin and GAPDH as the loading controls. Data show mean ± SD of two to three independent experiments. *p<0.0001. **c**: Immunofluorescence analysis showing filopodia formation (white arrows), accumulation of F-actin and its colocalization with FGD4 (white arrows) at the cell periphery in FGD4-EGFP expressing cells (panels 1, 3 and 5 from the top) compared to control cells, which was not altered upon treatment with ROCK inhibitor (ROCKi; compare panel 3 and panel 5 from the top). Nuclei were stained with DAPI. Scale bar: 10μm.

To understand FGD4 induced activation of CDC42/PAK pathway and actin reorganization, we monitored phospho-PAK concentration in the extracts of C4-2B cells expressing FGD4 using Western blots. Our results showed a significant increase in phospho-PAK (4.9-fold) in FGD4 expressing cells (Fig. 6a, b). It is known that CDC42 mediated PAK activation [41] leads to actin reorganization through activating phosphorylation of LIMK1 followed by inactivation of cofilin through phosphorylation [42]. Cofilin inactivation in turn, leads to accumulation of F-actin resulting in lamellipodia and filopodia formation, membrane protrusion and cell migration [43]. To examine the functional status of the downstream effector of CDC42/PAK, cofilin phosphorylation was determined next using Western blots. Our results showed a 2.0 -fold increase in phospho-cofilin levels in FGD4 expressing cells (Fig.6a and b). Additionally, changes in actin dynamics were monitored by immunofluorescence (IF) analysis of F-actin staining. Our analysis showed an increased accumulation of F-actin at the cell cortex and colocalization of FGD4 with F-actin in FGD4-EGFP positive cells. Appearance of filopodia was noted also in GFP positive cells expressing FGD4-EGFP (Fig. 6c). To determine the interference of Rho/ROCK in FGD4 induced actin reorganization, FGD4-EGFP expressing C4-2B cells were treated with ROCK inhibitor Y-27632 or DMSO before phalloidin staining. Inhibition of ROCK did not show any change in filopodia formation, cortical accumulation of F-actin or colocalization of FGD4 and actin (Fig. 6c).

### Inhibition of FGD4 expression improved drug sensitivity of prostate cancer cells

**Fig. 7:**
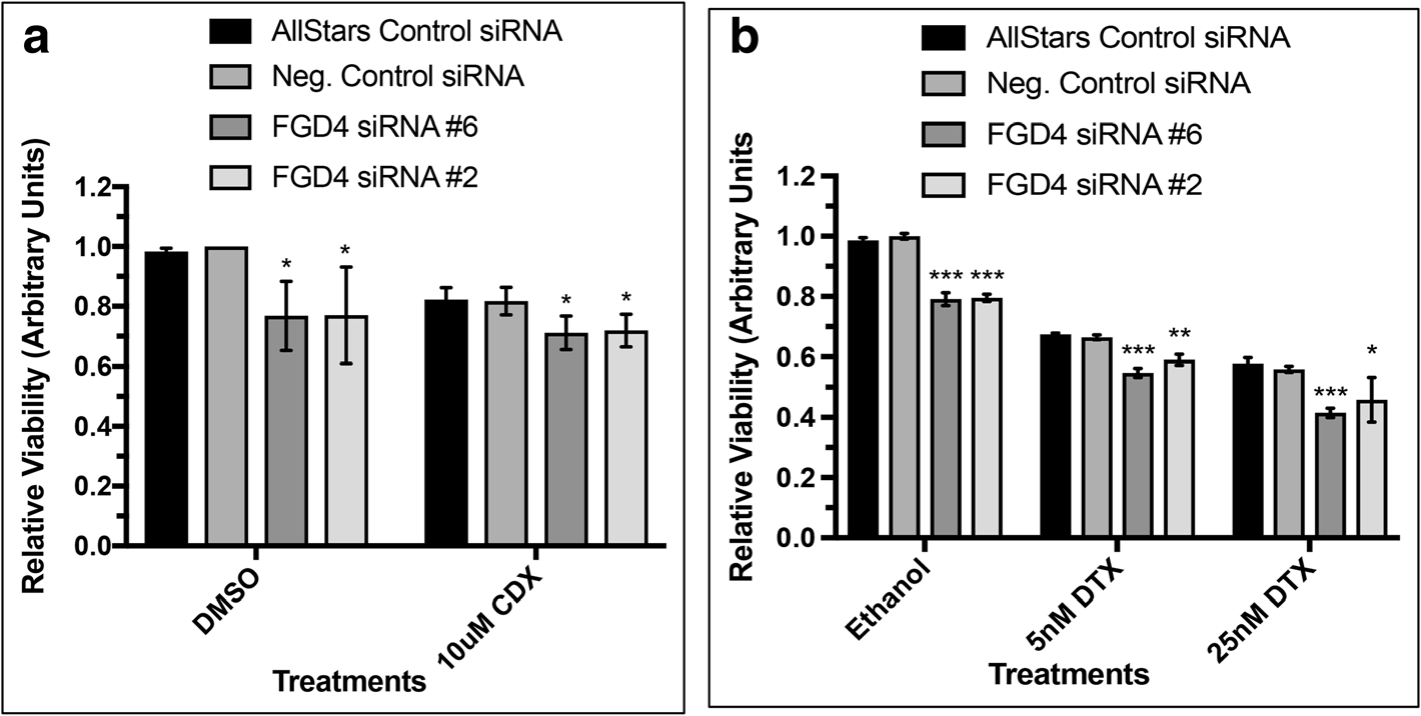
Knockdown of FGD4 increased drug sensitivity of prostate cancer cells. Prostate cancer cells. LNCaP-104S and PC-3 were transfected with FGD4 siRNA or control siRNAs and treated with Casodex (CDX) and docetaxel (DTX). Sensitivity of cells to specific treatments was measured through MTS assays. DMSO was used as the vehicle control. **a**: Percent reduction in viability of LNCaP-104S cells transfected with FGD4 siRNAs following treatment with 10μM CDX compared to cells transfected with control RNAs. Data represent mean ± SD of at least three separate experiments. *p<0.01 **b**: Percent reduction in viability of PC-3 cells transfected with FGD4 siRNA following treatment with 5 nM and 25 nM DTX compared to cells transfected with control RNAs. Data represent mean ± SD of three independent analyses. *p≤0.05, **p≤0.005, ***p≤0.005.

Our previous studies showed an up regulation of FGD4 in prostate cancer cells exhibiting resistance to Casodex, which could be the result of down regulation of miR-17-92a miRNA cluster expression that targets FGD4 through binding to the seed sequence at the 3’UTR [32]. In this study, we examined the relationship of FGD4 expression and sensitivity to chemotherapeutic agents that are currently being used in the clinic. We used transiently transfected LNCaP-104S with FGD4 siRNAs #2 and #6 or the control siRNAs and treated with Casodex (10 μM) for 48 h. Our results showed a 10–15% decrease in the viability of FGD4 siRNA transfected LNCaP-104S cells to the AR antagonist Casodex compared to the control siRNA (Fig. 7a). As noted earlier, inhibition of FGD4 reduced cell viability upon vehicle treatment in both LNCaP-104S and PC-3 cells compared to the control siRNA transfections. Inhibition of FGD4 expression also increased sensitivity to docetaxel treatment of PC-3 cells compared to the control siRNA transfection (Fig. 7b). An 8–15% decrease in the number of viable cells was noted upon docetaxel treatment of FGD4 siRNA transfected PC-3 cells compared to the control siRNA transfected cells. These results indicate that knockdown of FGD4 may have a potential therapeutic benefit for both androgen-dependent and androgen-independent prostate cancer cells.

## Discussion

In this study, we showed that FGD4 expression levels are increased in advanced prostate cancer compared to the luminal cells in benign prostatic hyperplasia. Additionally, we identified an important role of FGD4 in cell proliferation, cell cycle progression and cell migration, which is most likely mediated through activation of CDC42. We also showed the beneficial effect of inhibiting FGD4 expression on drug sensitivity.

Previous studies on FGD4 were mostly focused on its mutations and its association with demyelinating neuropathy in patients with CMT disease. A number of mutations resulting in premature termination of the protein and generation of a new donor splice site leading to truncated exon 7 and a frame shift have been detected in these patients [13]. A recent study showed that loss of FGD4 in Schwann cells reduced endocytosis through altered recruitment of activated CDC42 to the membrane, resulting in irregular accumulation of proteins at the plasma membrane and altered myelin membrane dynamics [2]. Nonetheless, limited information is available on the association of FGD4 with cancer. One study showed that the latent membrane protein (LMP) 1 of Epstein-Barr virus promotes increased motility of nasopharyngeal carcinoma (NPC) cells through increased CDC42 activity and remodeling of the actin cytoskeleton. The effect of LMP-induced increased motility is mediated through its direct interaction with FGD4, leading to enhanced GEF activity and thereby increasing activation of CDC42 [44]. Metastasis suppressor SSeCKs (AKAP12) regulates chemotaxis through modulating localization of chemotaxis regulators including Rac1 and CDC42. Loss of SSeCKs enriches membrane localization of FGD4 through its interaction with PIP2/3 via its PH and FYVE domains and with the actin filament via its FAB domain, which leads to localized activation of CDC42 [45].

In our earlier studies, we reported up-regulation of FGD4 expression in Casodex resistant prostate cancer cells, which is possibly the result of loss of expression of miR-17-92a cluster miRNAs in these cells. We further showed that FGD4 destabilization by miR-17-92a was through binding at the 3’UTR [36]. In this study, we provide evidence for the first time that FGD4 expression is upregulated in advanced prostate cancer with higher Gleason Scores and CRPC status. Our observation is supported by the TCGA database analysis showing FGD4 DNA copy number amplification and mRNA upregulation in a subset of neuroendocrine prostate cancer and in prostate carcinoma. Our results add another GEF in the growing list of GEFs that play important roles in prostate cancer and regulation of AR activity. One such GEF is VAV3, which is overexpressed in androgen independent prostate cancer cells and tissues, and up-regulates AR activity in prostate cancer cells [46].

Our results showed the dependence of prostate cancer cells on FGD4 expression for normal functioning, as down regulation of FGD4 reduced cell viability and led to G2/M arrest. The role of activated CDC42 in regulation of metaphase is well documented, specifically during spindle orientation [47], maintenance of centrosome integrity [48] and bi-orient attachment of spindle microtubules to kinetochores [49]. CDC42 is the primary Rho GTPase that participates in the chromosome alignment at the metaphase plate. Specific GEFs are identified that activate CDC42 during specific sub-stages of prometaphase/metaphase, which allows localized increase in the CDC42-GTP pool for activation of downstream effectors. Our results showing G2/M arrest upon FGD4 siRNA treatment suggests the requirement of FGD4, possibly as a CDC42 activator for progression of mitotic phases. However, the role of FGD4 in actin cytoskeleton reorganization through direct binding to actin filament [5] cannot be ruled out as modulation of dynamics of the cortical actin is essential during spindle orientation [50].

Another hallmark of cancer progression is enhanced cell migration. Being an actin binding protein and an activator of CDC42, FGD4 is expected to contribute in the regulatory mechanism of cell motility. In support of that, we noted a significant decrease (50%) in cell migration in scratch assays and a reduced activation of CDC42 upon inhibition of FGD4 expression in prostate cancer cells. This experiment was conducted without any chemotactic stimulus, which suggests that the inherent highly migratory nature of PC-3 cells requires an optimum level of FGD4. This assumption is further supported by our scratch assay results obtained upon ectopic expression of FGD4, which showed a significant increase (~ 55%) in cell migration. This observation is further supported by increased expression of mesenchymal marker SLUG and decreased expression of the epithelial marker E-cadherin in these cells, which suggests an indirect activation of EMT upon FGD4 overexpression. Furthermore, the function of FGD4 on modulating actin dynamics also plays an important role in promoting migratory behavior, which is through activation of CDC42/PAK/LIMK1 pathway as noted by increased activation of PAK and LIMK1 through increased phosphorylation of PAK and cofilin, a substrate of LIMK1. Activation of the CDC42/PAK pathway was further supported by our immunofluorescence analysis showing F-actin accumulation and filopodia formation at the cell periphery, which is not mediated by Rho/ROCK. Taken together, these observations establish FGD4 as a promoter of migratory behavior of advanced prostate cancer cells.

Our observations on the involvement of FGD4 in maintaining functional homeostasis of prostate cancer cells suggest its potential for being a suitable therapeutic target. Considering FGD4’s role in myelination, the possibility exists that general inhibition of FGD4 expression may lead to neuropathic effects. However, patients with CMT Disease Type 4H, in which FGD4 is absent or truncated, have very slow progression of peripheral nerve demyelination [2]. If used in treatment of prostate cancer, inhibition of FGD4 would be limited to short-term, localized therapy, compared to the sustained inactivation of FGD4 in Schwann cells of patients with CMT Type 4H, and is unlikely to have the same effects. FGD4 has been proposed as a paclitaxel-sensitizer gene, as silencing of FGD4 improved paclitaxel sensitivity of H1155 non-small cell carcinoma cells but this effect was not observed in any other cancer cell line [51]. Nonetheless, our results show an increased sensitivity of PC-3 cells to docetaxel and of androgen-sensitive LNCaP-104S cells to Casodex upon silencing of FGD4. It can be speculated that the effect of the microtubule stabilizing drug docetaxel on the mitotic arrest and induction of apoptosis could be aided by the interference in the G2/M progression upon silencing of FGD4. The effect of Casodex, on the other hand, has been shown to be mediated by inhibition of G1 phase progression through inactivating AR transcriptional activation [52, 53] and induction of apoptosis through caspase dependent and independent pathways [54]. It is possible that silencing of FGD4 can also have a synergistic effect on the Casodex mediated reduction in cell viability through its effect on G2/M arrest. However, the exact mechanistic role of FGD4 in cell cycle progression needs further study.

## Conclusion

Nonetheless, our results provide novel information on the association of FGD4 with aggressive prostate cancer and demonstrate a beneficial effect of inhibition of FGD4 in improving the effectiveness of therapeutic agents for both androgen dependent and castration resistant prostate cancer cells.

## Additional files

Additional file 1: Supplementary tables showing the STR profile of the cell lines and the source of antibodies used for this study. (TIF 1970 kb)
