# Supplementary figures and images for: Expression of FGD4 positively correlates with the aggressive phenotype of prostate cancer
Source: BMC Cancer. 2018 Dec 17;18:1257. doi: 10.1186/s12885-018-5096-9 (PMC6296060; doi:10.1186/s12885-018-5096-9)

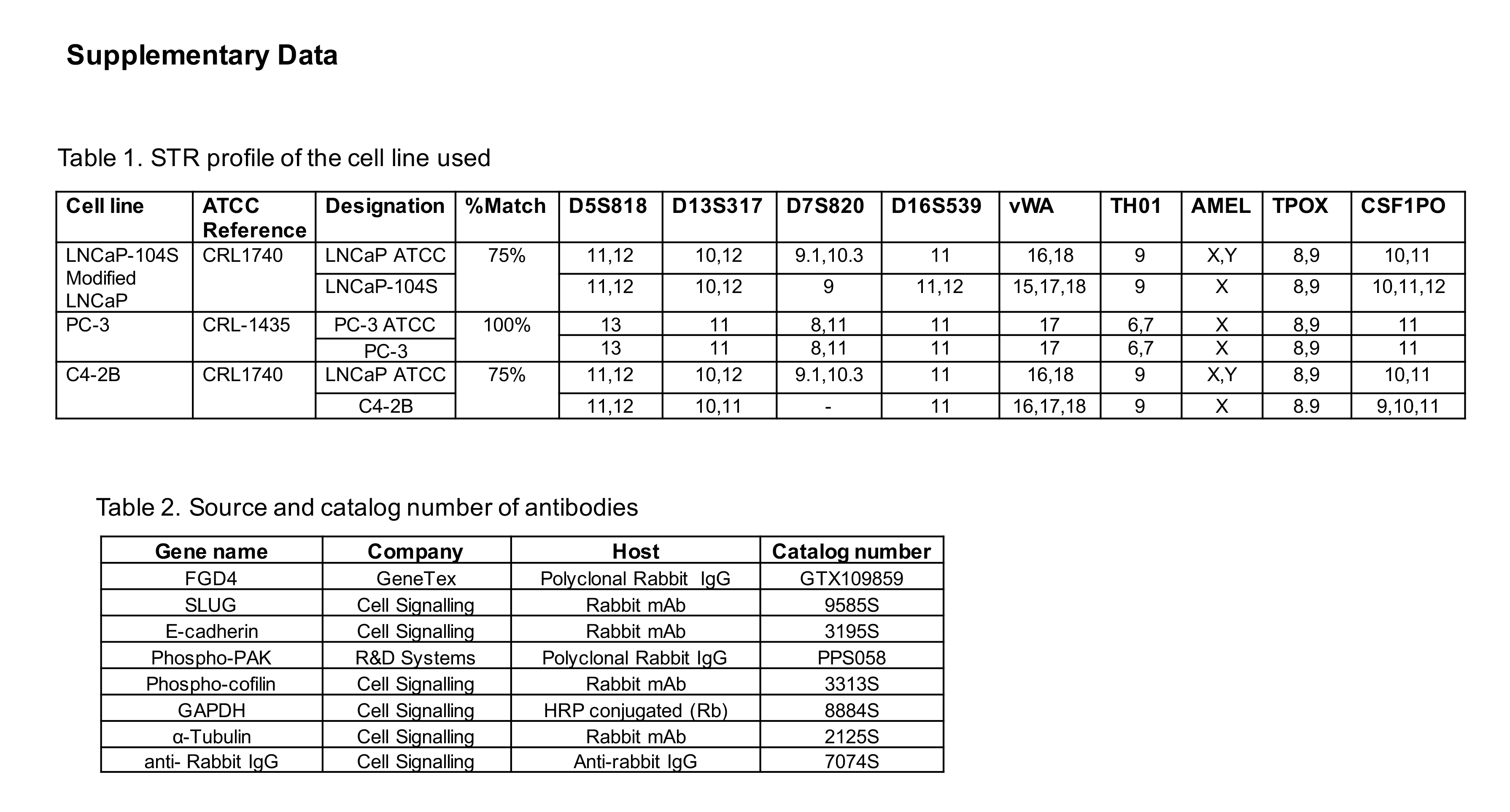

Supplement: Supplementary file 1 — Supplementary tables showing the STR profile of the cell lines and the source of antibodies used for this study. (TIF 1970 kb) [file 12885_2018_5096_MOESM1_ESM.tif]
